# Acyl ethanolamides in Diabetes and Diabetic Nephropathy: Novel targets from untargeted plasma metabolomic profiles of South Asian Indian men

**DOI:** 10.1038/s41598-019-54584-2

**Published:** 2019-12-02

**Authors:** Sarita Devi, Bajanai Nongkhlaw, M. Limesh, Roshni M. Pasanna, Tinku Thomas, Rebecca Kuriyan, Anura V. Kurpad, Arpita Mukhopadhyay

**Affiliations:** 1 Division of Nutrition, St. John’s Research Institute, St. John’s National Academy of Health Sciences, Bangalore, India; 2 Department of Nephrology, St. John’s Medical College and Hospital, St. John’s National Academy of Health Sciences, Bangalore, India; 3 Department of Biostatistics, St. John’s Medical College and Hospital, St. John’s Research Institute, St. John’s National Academy of Health Sciences, Bangalore, India

**Keywords:** Metabolomics, Diabetes complications

## Abstract

The pathophysiology of diabetic nephropathy (DN) in type 2 diabetes (T2D) patients is minimally understood. We compared untargeted high-resolution accurate mass (HRAM) orbitrap-based plasma metabolomic profiles of 31 T2D-DN (with estimated glomerular filtration rate ≤80 mL/min/1.73 m^2^), 29 T2D and 30 normal glucose tolerance (NGT) Indian men. Of the 939 plasma metabolites that were differentially abundant amongst the NGT, T2D and T2D-DN (ANOVA, False Discovery Rate – FDR adjusted *p-*value < 0.05), 48 were associated with T2D irrespective of the renal function of the subjects. Acyl ethanolamides and acetylcholine were decreased while monoacylglycerols (MAGs) and cortisol were elevated in both T2D and T2D-DN. Sixteen metabolites, including amino acid metabolites Imidazolelactate and N-Acetylornithine, changed significantly between NGT, T2D and T2D-DN. 192 metabolites were specifically dysregulated in T2D-DN (ratio ≥2 or ≤0.5 between T2D-DN and T2D, similar abundance in NGT and T2D). These included increased levels of multiple acylcarnitine and amino acid metabolites. We observed a significant dysregulation of amino acid and fatty acid metabolism in South Asian Indian male T2D-DN subjects. Unique to this study, we report a reduction in acyl ethanolamide levels in both T2D and T2D-DN males. Those with dysregulation in acyl ethanolamides, which are endogenous agonists of GPR119, are likely to exhibit improved glycemic control with GPR119 agonists.

## Introduction

The global prevalence of T2D is estimated to be 9% among adults^1^. Individuals of South Asian descent seem especially at high risk^2^. Even more disconcerting is the possibility of a more aggressive course of the underlying pathophysiology of the disease in South Asian Indians as underlined by the recent findings in Chennai, India, of one of the highest rates of conversion from pre-diabetes to T2D^3^. It is still not clear what specific metabolic dysregulations underlie this susceptibility or aggressive course.

Diabetic nephropathy (DN) is the leading cause of end stage renal disease (ESRD) worldwide^4^. A 2% prevalence of overt nephropathy and a 27% prevalence of microalbuminuria has been reported amongst South Asian Indian T2D patients in India^5^. The prevalence of DN is higher in Asians, African Americans and Native Americans compared to Caucasians, raising the possibility of a higher susceptibility of some populations to DN^6^. Only a subset of T2D patients (about 40%) develop DN and the progression from DN to ESRD is not uniform^7^, further emphasizing the heterogeneity of the pathophysiology of DN.

Strong correlations have been reported between insulin resistance and dysregulated lipid and amino acid metabolism^8,9^. Some of the primary metabolic coupling factors that might affect beta-cell function include glutamate, long chain acyl-CoA and diacylglycerol^10^. Further, metabolites associated with branched chain amino acid (BCAA) catabolism such as 3-hydroxy Isovalerate, 2-Ethyl 3-Hydroxy Propionate, Hydroxy Propionic acid as well as sphingolipid metabolism have been reported to be dysregulated in DN patients^11,12^.

Except for the prospective follow-up studies tracking the progression of T2D to DN^13^, most studies have compared metabolomic profiles directly between DN and healthy controls without comparing uncomplicated T2D metabolomic profiles, or have not specifically looked at metabolites associated with T2D and progression to DN in South Asian Indian subjects or have not adjusted their findings for the sex of the subjects. In this backdrop, we have compared untargeted plasma metabolomic profiles of well-characterized South Asian Indian male normal glucose tolerance (NGT), T2D and T2D-DN subjects with the goal of identifying metabolites that are common or different between T2D and T2D-DN in this high-risk population. In this hypothesis generating study, data on such metabolites and the associated biochemical pathways will form the basis for testable hypotheses for better understanding of the pathophysiology of T2D and T2D-DN toward elucidation of novel targets for prevention as well as treatment.

## Results

Socio-demographic characteristics, anthropometric measurements, metabolic profiles and dietary intakes of the study participants are summarized in Table 1. BMI, body fat%, fat mass, appendicular lean mass and dietary intakes (energy and macronutrients) were similar between the diabetic (T2D and T2D-DN) and NGT control groups. The diabetic groups were older, had higher waist-hip ratio and higher fasting glucose (>120 mg/dL) and HbA1c (>6.5%). Systolic blood pressure and serum creatinine levels were higher in the T2D-DN group.

**Table 1:** Subject characteristics, metabolic profile and dietary intake of the 90 study subjects.

	NGT	T2D	T2D-DN	*P* value
n	30	29	31	
Age (years)	38.2 ± 5.7^a^	48.8 ± 8.1^b^	52.8 ± 7.2^b^	<0.001
Height (cm)	168.3 ± 8.2	165.9 ± 5.0	166.0 ± 6.4	0.281
Weight (kg)	71.2 ± 8.8	68.0 ± 9.3	67.6 ± 8.1	0.229
BMI (kg/m^2^)	25.1 ± 2.2	24.7 ± 2.8	24.5 ± 2.4	0.659
Waist-Hip Ratio (cm)	0.91 ± 0.05^a^	0.96 ± 0.06^b^	0.98 ± 0.08^c^	<0.001
Years since diagnosed as T2D^*^	0.0 (0.0, 0.0)^a^	3.0 (1.0, 12.3)^b^	10.0 (5.8, 15.5)^b^	<0.001
Systolic blood pressure (mmHg)	121.0 ± 11.7^a^	128.8 ± 16.2^a^	141.9 ± 27.3^b^	<0.001
Diastolic blood pressure (mmHg)	81.4 ± 8.0	83.3 ± 8.5	87.3 ± 15.1	0.117
Body fat (%)	30.9 ± 4.8	29.5 ± 5.2	29.0 ± 6.3	0.384
Fat mass (kg)	21.3 ± 5.3	19.5 ± 5.3	19.2 ± 5.5	0.257
Android Fat (%)	42.8 ± 5.1	41.2 ± 6.1	40.2 ± 7.1	0.250
Gynoid Fat (%)	34.7 ± 4.8^a^	31.1 ± 5.0^b^	29.8 ± 6.^b^	0.002
Android:Gynoid ratio	1.24 ± 0.15^a^	1.34 ± 0.16^a^	1.36 ± 0.18^b^	0.014
Appendicular lean mass (kg)	47.0 ± 5.0	45.8 ± 5.2	45.8 ± 4.5	0.572
Bone Mineral Content (kg)	2.9 ± 0.4^a^	2.6 ± 0.3^a,b^	2.6 ± 0.4^b^	0.020
**Metabolic profile**^**†**^				
HbA1c (%)	5.2 ± 0.2^a^	7.9 ± 1.6^b^	7.9 ± 1.9^b^	<0.001
Fasting Glucose (mg/dL)	85.9 ± 7.5^a^	153.9 ± 68.3^b^	135.9 ± 67.2^b^	<0.001
Fasting Insulin (mU/L)^#^	10.2 ± 5.8	11.6 ± 6.8	17.7 ± 20.7	0.070
C-Peptide (ng/mL)	2.6 ± 0.9^a^	3.9 ± 1.9^a^	4.7 ± 3.0^b^	0.001
Cholesterol (mg/dL)	183.2 ± 31.7	194.7 ± 44.6	171.7 ± 64.0	0.195
HDL Cholesterol (mg/dL)	42.7 ± 7.1	39.2 ± 7.6	38.9 ± 8.0	0.109
LDL Cholesterol (mg/dL)	122.4 ± 27.1	123.7 ± 34.4	107.0 ± 45.9	0.151
Triglycerides (mg/dL)	120.1 ± 52.8^a^	181.8 ± 89.1^b^	172.0 ± 122.9^a^	0.027
Creatinine (mg/dL)	0.9 ± 0.1^a^	0.9 ± 0.2^a^	2.1 ± 1.5^b^	<0.001
**Dietary intake**^**^**^				
Energy (kcal)	1534 (1392, 1667)	1577 (1269, 1907)	1325 (1043, 1709)	0.126
Protein (g)	47.2 (38.4, 55.6)	51.2 (40.9, 71.1)	41.7 (30.6, 54.1)	0.111
Protein energy%	11.8 (10.0, 13.7)	12.5 (11.7, 16.5)	12.1 (10.9, 14.0)	0.112
Carbohydrate (g)	247.3 (213.9, 277.9)	235.0 (173.0, 305.0)	213.5 (168.1, 270.5)	0.091
Carbohydrate energy%	66.4 (61.6, 69.2)	61.0 (51.5, 70.6)	62.7 (59.6, 67.5)	0.297
Fat (g)	37.1 (30.9, 48.0)	43.4 (28.4, 52.9)	32.2 (25.2, 50.3)	0.489
Fat energy%	22.7 (19.7, 26.7)	24.1 (18.3, 29.3)	24.4 (20.0, 28.9)	0.659
Saturated fat (g)	11.8 (8.9, 17.8)	12.3 (8.2, 18.5)	13.1 (5.9, 19.0)	0.813
Palmitic acid (g)	4.85 (3.54, 6.30)	5.10 (3.38, 6.57)	4.13 (2.83, 5.60)	0.266
Steric acid (g)	2.13 (1.59, 3.07)	2.38 (1.46, 3.22)	1.83 (1.22, 2.80)	0.354
Monounsaturated fat (g)	7.91 (7.52, 13.67)	9.60 (5.95, 14.3)	8.0 (5.7, 11.7)	0.559
Palmitoleic acid (g)	0.21 (0.14, 0.33)	0.29 (0.13, 0.37)	0.20 (0.11, 0.33)	0.379
Oleic acid (g)	7.40 (6.76, 13.09)	8.91 (5.45, 13.4)	7.15 (5.20, 10.17)	0.368
Polyunsaturated fat (g)	14.4 (10.8, 17.9)	13.4 (8.8, 17.4)	13.6 (8.4, 17.0)	0.818
Linoleic acid (g)	14.0 (10.5, 17.5)	13.0 (8.5, 17.0)	13.5 (8.2, 16.6)	0.782
Linolenic acid (g)	0.30 (0.25, 0.36)	0.29 (0.20, 0.39)	0.26 (0.19, 0.34)	0.474
Arachidonic acid (g)	0.000 (0.000, 0.010)^a^	0.002 (0.000, 0.081)^a,b^	0.001 (0.001, 0.011)^b^	0.031

Data presented as mean ± SD, Labelled means in a row without a common superscript letter differ by post-hoc Tukey’s test, *P* < 0.05.

*Data presented as median (quartile1, quartile3).

Labelled medians in a row without a common superscript letter differ by post-hoc Dunn test, Bonferroni adjusted for multiple testing *P* < 0.05.

^†^Blood was drawn from fasting volunteers.

^#^Value for n = 89 (NGT^:^ n = 29).

^^^Data calculated from one 24-hour recall of dietary intake.

**Figure 1:**
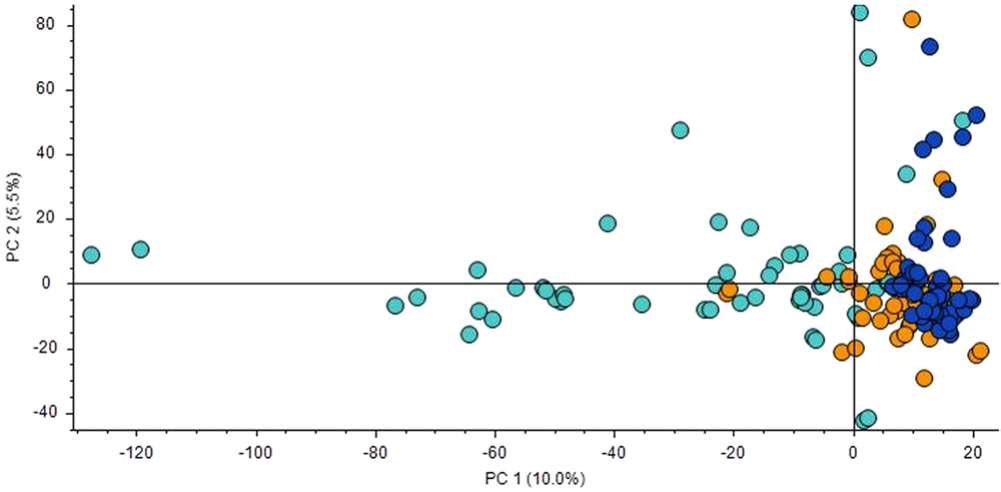
Multivariate analysis of untargeted metabolomics data using 5781 metabolites. Principal component analysis (PCA) plot of principal component (PC)1 and PC2 for each of the 90 plasma samples from subjects of normal glucose tolerance (NGT: dark blue circles), type 2 diabetes mellitus (T2D: orange circles) and T2D-Diabetic nephropathy (T2D-DN: light blue circles) groups.

For each fasted plasma sample, 5781 metabolite features were detected, of which 1763 could be assigned identities. Based on PCA, the 3 subject groups could be separated along PC1 (Fig. 1). PC1 and PC2 explained 10.0% and 5.5% respectively of the variance in the metabolomic data.

On further conducting univariate analyses by ANOVA, we observed that 939 metabolites were differentially abundant amongst the NGT, T2D and T2D-DN groups. In order to specifically identify the metabolites associated with the T2D and T2D-DN, we further conducted systematic pathway analysis on these 939 metabolites.

### Metabolites significantly dysregulated between NGT, T2D and T2D-DN

**Figure 2:**
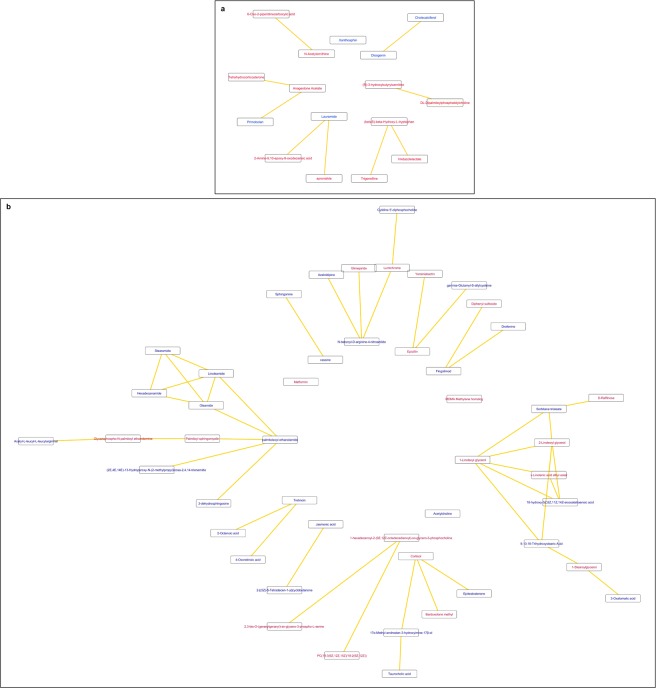
Biochemical pathway and chemical relationships network of (**a**) the 16 metabolites significantly dysregulated between NGT and T2D, T2D and T2D-DN as well as NGT and T2D-DN (FDR adjusted *p-*value <0.05) and (**b**) the 48 metabolites significantly associated with T2D irrespective of renal function of the subjects [significantly dysregulated (FDR adjusted *p-*value <0.05) between NGT and T2D and between NGT and T2D-DN but not between T2D and T2D-DN]. Blue represents downregulated and red represents upregulated metabolites in the diabetic groups (T2D and T2D-DN) compared to NGT. (NGT: normal glucose tolerance, T2D: type 2 diabetes mellitus, T2D-DN: type 2 diabetes mellitus-diabetic nephropathy).

**Figure 3:**
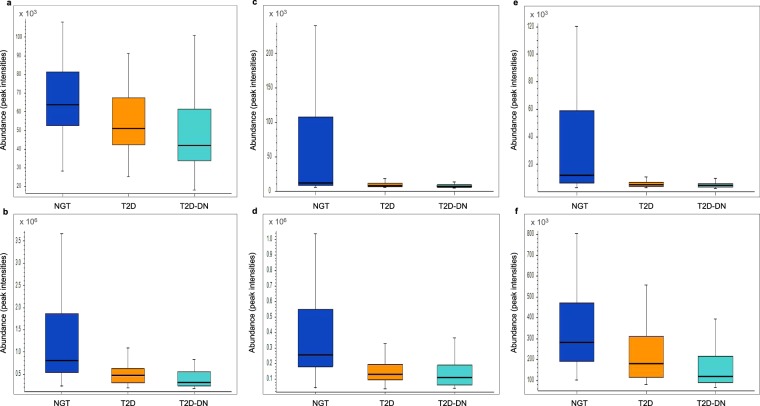
Box plots depicting levels of (**a**) Hexadecanamide, (**b**) Lauramide (**c**) Linoleamide, (**d**) Oleamide, (**e**) Palmitoleoyl ethanolamide and (**f**) Stearamide in normal glucose tolerance (dark blue), type 2 diabetes mellitus (orange) and type 2 diabetes mellitus-diabetic nephropathy (light blue) subjects.

To identify metabolites significantly elevated or reduced in both T2D and T2D-DN compared to the NGT as well as dysregulated between T2D and T2D-DN in the same direction, we selected metabolites that were significantly dysregulated between NGT and T2D, between T2D and T2D-DN as well as between NGT and T2D-DN (FDR adjusted *p-*value <0.05). Of 36 such metabolites identified, 16 could be mapped to biochemical pathways using CytoScape (Supplementary Table 1, Fig. 2a). We observed an increase in amino acid metabolites such as Imidazolelactate (Supplementary Fig. 1a), N-Acetylornithine and 6-Oxo-2-piperidinecarboxylic acid as well as in (R)-3-hydroxybutyrylcarnitine. Lauramide and cholecalciferol levels declined, by 20% and 30% in T2D compared to NGT and by 18% and 24% in T2D-DN compared to T2D, respectively (Fig. 3a, Supplementary Fig. 1b).

### Metabolites specifically dysregulated in T2D and T2D-DN irrespective of renal function

We deemed metabolites that were significantly (FDR adjusted *p-*value <0.05) dysregulated between NGT and T2D and between NGT and T2D-DN but were similar between T2D and T2D-DN groups to be associated with T2D irrespective of the renal function. Of 208 such metabolites identified, we could map 48 to distinct biochemical pathways (Supplementary Table 2, Fig. 2b). Monoacylglycerols (MAGs) 1-Stearoylglycerol (Supplementary Fig. 1c), 1-Linoleoyl glycerol and 2-Linoleoyl glycerol were elevated while acyl ethanolamides such as palmitamide (hexadecanamide), linoleamide, oleamide, palmitoleoyl ethanolamide and stearamide (Fig. 3b–f) were all decreased in T2D and T2D-DN compared to NGT with similar levels between T2D and T2D-DN groups. Cortisol and few phosphatidylcholine species, such as 1-hexadecanoyl-2-(9Z,12Z-octadecadienoyl)-sn-glycero-3-phosphocholine were elevated while acetylcholine was decreased in T2D and T2D-DN. As expected, drugs such as metformin and glimepiride were elevated in T2D and T2D-DN.

### Metabolites specifically dysregulated in T2D-DN

To identify metabolites specifically associated with T2D-DN, we selected metabolites that were significantly dysregulated between NGT and T2D-DN as well as between T2D and T2D-DN (FDR adjusted *p-*value <0.05) but not between NGT and T2D. This constituted the largest group of 1447 metabolites, of which 501 could be mapped to biochemical pathways. To reduce the complexity of the resulting pathway map and to focus on the metabolites with relatively higher effect sizes, we further mapped 192 of the 501 metabolites that exhibited a difference in ratio of ≥2 or ≤0.5 between the T2D-DN and T2D groups (Supplementary Table 3, Supplementary Fig. 2). Multiple amino acids and their metabolites were elevated in T2D-DN. Amongst the amino acids, methionine exhibited the largest increase (12.5 fold higher compared to NGT and 6.9 folds higher compared to T2D). The n-6 and n-3 fatty acid pathways were dysregulated, with increase in arachidonic acid and docosahexaenoic acid ethyl ester and decrease in docosapentaenoic acid. We also observed dysregulation in the carnitine-fatty acid metabolism pathway, with elevated levels of DL-Carnitine, O-3-methylglutarylcarnitine, acetyl-L-carnitine, propionylcarnitine, hydroxypropionylcarnitine and hexanoylcarnitine while levels of palmitoylcarnitine (Supplementary Fig. 1d) and 2-methylbutyrylcarnitine were reduced. As expected, creatinine, multiple medication and medication-related metabolites were increased exclusively in T2D-DN. The choline-phosphatidylcholine pathway was dysregulated in T2D-DN with reduced levels of oleoyl-lysophosphatidylcholine, 1-Hexadecanoyl-sn-glycero-3-phosphocholine and L-alpha-Glycerylphosphorylcholine and elevated levels of 1-tetradecanoyl-2-[(9Z)-octadecenoyl]-sn-glycero-3-phosphocholine. Choline levels were 2.3 and 1.9 folds higher in T2D-DN compared to NGT and T2D respectively (Supplementary Fig. 1e).

As the metabolites observed to be dysregulated in T2D-DN are likely to be associated with renal function, we tested and observed within the T2D-DN group, significant association between renal function, expressed as estimated Glomerular Filtration Rate (eGFR), and abundance of the 20 metabolites with the lowest FDR adjusted *p-*value in difference in abundance between T2D and T2D-DN (Supplementary Table 4).

### Metabolites associated specifically with T2D

Only 3 metabolites, 1-arachidonoyl-sn-glycero-3-phosphocholine, phenylalanylproline and 4-(8-Methyl-8,9-dihydro-7H-[1,3]dioxolo[4,5-h][2,3]benzodiazepin-5-yl)aniline were significantly dysregulated between T2D and NGT and between T2D and T2D-DN (FDR adjusted *p-*value <0.05) but were similar in abundance between NGT and T2D-DN (Supplementary Table 5, Supplementary Fig. 1f–h).

## Discussion

We have analysed untargeted plasma metabolomic patterns associated with T2D and T2D-DN to parse plasma metabolites into those that are associated with T2D irrespective renal involvement and those associated specifically with T2D-DN, in Indian men, a high-risk population whose T2D and T2D-DN associated metabolomic profile has not been reported.

Unique to our study, we observed consistent reduction of multiple species of acyl ethanolamides in both T2D and T2D-DN fasting plasma. *N*-acylphosphatidylethanolamine (*N*-APE), the precursor of acyl ethanolamides^14^, as well as acyl ethanolamides such as oleamide, have been implicated as small intestine-derived circulating factors synthesized in response to ingested fat, that promote satiety and inhibit food intake by activating the nuclear receptor PPAR-α^15^. Since we exclusively analysed fasted plasma samples, and dietary intakes of energy or macronutrients were similar between the control and diabetic groups, it is unlikely that role of acyl ethanolamides in T2D-associated dysglycemia could be mediated by regulation of feeding. Further supporting this argument, intestinal epithelia-specific mouse knockout of *N*-acylphosphatidylethanolamine phospholipase D (*N*-APE-PLD), the enzyme catalysing conversion of *N*-acylphosphatidylethanolamines to acyl ethanolamides, results in exacerbation of high fat diet-induced obesity and hepatic steatosis but does not affect glucose metabolism^16^ whereas adipocyte-specific *N*-APE-PLD knockout mice exhibit whole body fasted hyperglycemia and hyperinsulinemia, as well as hepatic and skeletal muscle but not adipose insulin resistance^17^.

**Figure 4:**
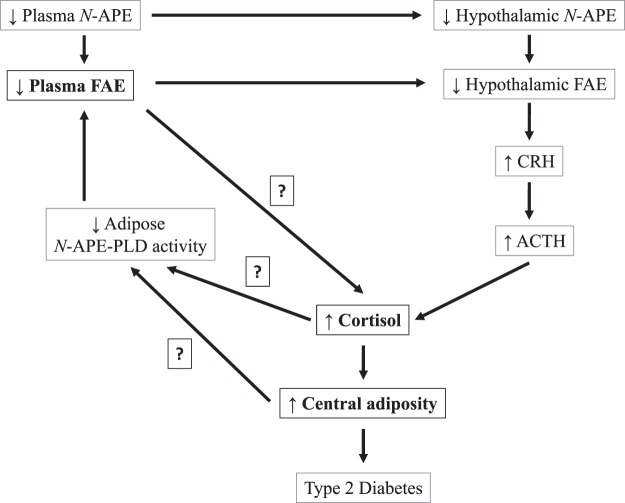
Framework for understanding the role of fatty acyl ethanolamides (FAEs) in precipitating T2D-associated dysglycemia. Lower plasma *N*-APE levels could lead to lower hypothalamic *N*-APE levels, upregulating the hypothalamic-pituitary-adrenal (HPA) axis, leading to increased plasma cortisol levels, central adiposity and development of dysglycemia associated with T2D. Proposed steps stemming from the findings of the current study are highlighted in bold while those lacking available evidence are highlighted with question marks. *N*-APE: *N*-acylphosphatidylethanolamine, *N*-APE-PLD: *N*-acylphosphatidylethanolamine phospholipase D, CRH: Corticotropin-releasing Hormone, ACTH: Adrenocorticotropic Hormone.

Low plasma acyl ethanolamide levels in the T2D and T2D-DN groups could result in low brain acyl ethanolamide levels as both radiolabelled *N*-APE^14^ and palmitoylethanolamide can cross the rat blood-brain barrier and concentrate in the hypothalamus^18^. In the paraventricular hypothalamic nucleus (PVN), the acyl ethanolamides and the cannabinoid signalling system could downregulate the hypothalamic-pituitary-adrenal (HPA) axis, as oral treatment of lean and obese Zucker rats with the cannabinoid-1 receptor (CB_1_) antagonist rimonabant, an anorectic drug, leads to increase in basal corticosterone levels^19^, similar to the higher fasting plasma cortisol levels that we observed in the T2D and T2D-DN groups. Significant, positive associations between morning, fasting serum cortisol and fasting glucose concentrations and insulin resistance in 509 subjects from Mysore, India, with 15% prevalence of T2D have been reported earlier^20^. Persistent activation of the HPA axis, therefore, could likely precipitate the T2D-associated dysglycemia via increase in central adiposity,^21^ evident in the T2D and T2D-DN groups based on higher ratio of Android:Gynoid fat% and waist-hip ratio. Based on our findings and associated relevant findings in humans and animal models summarized above, we hypothesize that lower plasma *N*-APE levels could lead to lower hypothalamic *N*-APE levels, upregulating the hypothalamic-pituitary-adrenal (HPA) axis, leading to increased plasma cortisol levels, central adiposity and development of dysglycemia associated with T2D (Fig. 4).

Apart from their cannabinoid-signaling related effects on dysglycemia, palmitoleoyl ethanolamide could also be acting as a potent lipid agonist of GPR119^22^, sensing lipids in the intestinal enteroendocrine cells, stimulating glucagon-like peptide-1 (GLP-1) release as well as in the pancreatic β-cells, and enhancing glucose-dependent insulin secretion^23^. Though many GPR119 agonists have been recently tested as a novel class of antidiabetic drugs relying on such data from *in vitro* and animal studies, none have progressed beyond phase II trials^24^ except DS-8500a, that was reported to significantly improve glycemic control in a randomized, double-blind phase II trial in Japanese diabetic patients^25^. Considering there are no epidemiological data available on plasma levels of acyl ethanolamide, or endogenous agonists of GPR119, it is likely that the synthetic agonists are possibly going to be effective in improving glycemic control in ethnic populations where the endogenous GPR119 agonists are dysregulated in diabetic patients, such as in our study.

Multiple MAGs were consistently elevated in our diabetic study population (both T2D and T2D-DN, with no further dysregulation between T2D and T2D-DN). We postulate that such metabolites highlight T2D-associated dysglycemia with minimal or no involvement in diabetic nephropathy. As such, these metabolites are also likely to have prospective prognostic potential owing to their causal involvement in T2D-associated dysglycemia. MAGs such as 1-Palmitoylglycerol have been reported^26^ as positive contributors to Metabolomic Risk Scores discriminating between T2D and control fasting plasma from middle-aged European participants at baseline, implicating higher ‘fasting’ levels of MAGs in normoglycemic individuals as a sign of on-going or impending exhaustion of β-cell function/mass as MAGs enhance glucose-stimulated insulin secretion (GSIS) in the presence of glucose, in rat insulinoma cells, rat islets and human islets^27^.

We also observed significantly reduced levels of acetylcholine in T2D and T2D-DN plasma. This has been reported earlier, where a reduction of 50% in the risk of incident T2D of the highest quartile of plasma acetylcholine was observed in Chinese adults^28^, confirming the power of the present untargeted cross-sectional plasma metabolomic approach in identifying plasma metabolites likely to have predictive role in development of dysglycemia. Similar prospective studies from India have not yet been reported. We did not detect or quantitate glucose in our untargeted plasma metabolomics dataset as the analysis of polar metabolites requires pre-column derivatization (such as silylation before analysis by GCMS) or specialized chromatography^29–31^.

Very few metabolites exhibited a step-wise dysregulation between NGT, T2D and T2D-DN groups. The progressive increase in levels of amino acid metabolites such as Imidazolelactate and N-Acetylornithine are likely to be reflective of the role of kidney in amino acid metabolism^32^, and this needs to be confirmed in prospective studies. We also observed a progressive decline of cholecalciferol in T2D and T2D-DN, which is expected in light of the widely recognized renoprotective role of vitamin D^33^.

The T2D-DN specific metabolites formed the largest category of dysregulated metabolites, primarily due to the varied drug regimens the T2D-DN patients were on. Amino acids such as methionine, proline, leucine, tyrosine, glutamine, glutamic acid and their derivatives were uniformly increased in T2D-DN, which converges with the role of the kidney in amino acid metabolism, as well as earlier reports on plasma metabolomic profiles in patients with kidney dysfunction^34^. In addition, we also observed an elevation of plasma choline levels in T2D-DN subjects. Choline has been recently reported to be one of the 24 plasma metabolites associated negatively with HOMA-IR in the PREDIMED study^35^.

We observed significant dysregulation of the carnitine-fatty acid metabolism in T2D-DN subjects. A systematic review and meta-analysis of carnitine supplementation in ESRD patients reported a significant reduction of serum low-density lipoprotein (LDL), C-reactive protein (CRP) and associated reduction in inflammation, further linking this to lower cardiovascular complications and all-cause mortality in these patients^36^. L-carnitine is well known for its role as a protectant against cellular oxidative stress from various sources including lipid peroxidation^37^. Overall, the reduced levels of long-chain acylcarnitines combined with increased levels of short-chain acylcarnitines as well as of DL-carnitine in the T2D-DN subjects from our study suggest an impaired rate of β-oxidation, which has been recently reported to worsen with advancing chronic kidney disease (CKD)^38^.

We observed significant association between renal function, expressed as eGFR, and abundance of the 20 metabolites with the lowest FDR adjusted *p-*value in difference in abundance between T2D and T2D-DN, within the T2D-DN group. For instance, higher plasma imidazolelactate abundance was associated with lower eGFR values, both within the T2D-DN and T2D groups. This is expected as imidazolelactate is a non-metabolizable catabolite of histidine in humans^39^ and therefore, plasma abundance of imidazolelactate could be acting as an indicator of renal excretory potential. The endogenous metabolites that exhibit significant association with renal function can potentially be utilized to track renal function once validating prospective studies are conducted to confirm our observations.

The strength of this study is the utilization of a logical data analysis plan in three groups of subjects, through which untargeted plasma metabolomic profiles were parsed toward identifying metabolites that first, are dysregulated between NGT and T2D subjects irrespective of renal function; second, are dysregulated in a step-wise manner between NGT, T2D and T2D-DN subjects, thereby likely to be associated with worsening kidney function in T2D-DN subjects and third, are dysregulated specifically in T2D-DN subjects, that included most of the myriad medications that the T2D-DN subjects consume. Further, to the best of our knowledge, we have described these plasma metabolomic patterns for the first time in South Asian Indians, a high-risk group, living in India, a country going through economic and nutrition transition^40^. We also intentionally restricted our study to plasma samples to men to avoid the probability of sex-specific metabolites masking metabolite perturbations associated with T2D and T2D-DN, as sex-specific differences in plasma metabolomic profiles is well documented^41–43^. Potential weaknesses of our study include the relatively small sample size and utilization of a single untargeted metabolomic platform and a single type of body fluid for metabolomic analyses. Also, difference in levels of metabolites between NGT and T2D as well as T2D-DN groups could potentially be explainable by the higher age of the T2D and T2D-DN groups compared to the NGT group as levels of a fraction of plasma metabolites have been reported by various groups to be associated with age^44,45^. For instance, elevated levels of cortisol in T2D and T2D-DN in our study could be attributed to age as plasma cortisol levels have been reported to be positively associated with age in both males and females^46^. Nevertheless, Fanelli *et al*. did not find plasma palmitoylethanolamide, oleoylethanolamide and anandamide levels to be associated with age in 144 Italian men, in the only study to date that has analysed association of these metabolites with age in human plasma^47^. Further, activity of brain and heart *N*-acylphosphatidylethanolamine phospholipase D (*N*-APE-PLD), the enzyme catalysing conversion of *N*-acylphosphatidylethanolamines to acyl ethanolamides, has been reported to increase ~15 folds from neonatal stage to adulthood in male Wistar rats while that of liver *N*-APE-PLD stayed unchanged with increasing age^48^, making it mechanistically unlikely that the that the reduced levels of multiple acyl ethanolamide species that we have observed in the T2D and T2D-DN plasma is due to their decline with age.

In summary, our findings suggest that the downregulation of acyl ethanolamides is a likely novel mechanism of T2D associated dysglycemia. Carnitine-fatty acid and amino acid metabolic pathways exhibit progressive dysregulation from normoglycemia to T2D and further to diabetic nephropathy. Prospective studies in both men and women can test the causal role of these metabolites in the precipitation of dysglycemia associated with T2D and in perturbed renal function in T2D-DN. These metabolites also provide fertile avenues for future mechanistic studies to identify on one hand, novel modifiable lifestyle changes for prevention and on the other, novel therapy targets for treatment of T2D and T2D-DN.

## Methods

### Study design and inclusion criteria

The study sample consisted of control (NGT), T2D and T2D-DN men who were recruited through the Department of Nephroplogy and the Nutrition and Lifestyle Clinic at St. John’s Medical College and Hospital, Bangalore.

Subjects included were in the age group 18–60 years, who consented to participate in the study, and were recruited according to American Diabetes Association (ADA) criteria in the following 3 groups: a) T2D subjects with DN (T2D-DN, n = 31) with an estimated glomerular filtration rate (eGFR, Modification of Diet in Renal Disease equation) ≤80 mL/min/1.73 m^2^); b) T2D subjects without diabetic nephropathy (T2D, n = 29); and c) Control subjects with normal glucose tolerance (NGT, n = 30) of age, sex and BMI similar to that of the case group subjects.

Subjects were excluded if their age was outside the range of 18–60 years, unwilling to participate in the study, participating in any other study and those who tested positive for hepatitis (HBsAg) and HIV. Those who had serious pre-existing medical conditions or required chronic or daily medical therapy (connective tissue diseases, inflammatory bowel disease, active tuberculosis, symptomatic heart disease) were excluded.

### Ethical approval and informed consent

The Institutional Ethics Committee of St. John’s Medical College and Hospital, Bangalore approved the study. The study protocol was explained in the local language of the participants and their signed, informed consent was obtained at recruitment. The study protocol was carried out in accordance with relevant guidelines and regulations.

### Socio-demographic data and medical history

Detailed socio-demographic data was collected by trained personnel using a well-structured questionnaire. The questionnaires was provided and explained in English or a local language in which the subject is most comfortable. A detailed clinical examination was performed for all subjects to ensure that subjects met inclusion criteria, and a blood and urine workup was included to evaluate liver and renal function. Participants were also asked for information regarding the medications and supplements they were currently taking, and verified from prescriptions that they were using.

### Anthropometric, blood pressure and pulse measurements

Subjects were weighed in minimal clothing using a digital scale, to a precision of 0.1 kg. The height of the subjects were recorded to the nearest 0.1 cm. Waist and hip measurements were also taken to estimate the Waist to hip ratio (WHR). Blood pressure and pulse was measured and recorded by trained staff after a relaxation period. Whole body and regional body composition was assessed by Dual-energy X-ray absorptiometry (DXA; DPXMD 7254, Lunar Corporation, Madison, WI). Total body fat (BF) was measured and expressed as a percentage of body weight (% BF).

### Sample Collection, clinical chemistry

Ten mL of blood was collected in EDTA (BD Vacutainer®, Becton, Dickinson and Company, Franklin Lakes, NJ) tubes between ∼0900 and 0930 hours by arm venepuncture after an overnight fast, and immediately transferred to an ice box until further processing. Samples were centrifuged within 1.5 hours of collection at 117 rcf for 10 minutes in a cold centrifuge (4 °C; REMI C-23 BL Cooling centrifuge, Mumbai, India), after which the plasma was separated, aliquoted in cryovials and stored at −80 °C until analysis.

Standard plasma and serum clinical chemistry assays on fasted samples included glucose, total cholesterol, HDL cholesterol, LDL cholesterol, triglycerides and creatinine (Beckman Coulter AU480 Chemistry Analyzer, Beckman Coulter, Brea, CA), insulin and C-peptide (ROCHE Hitachi Elecsys 2010 Chemistry Analyzer, Basel, Switzerland) and hemoglobin A1c (Siemens Dimension XPand Plus Analyzer, Siemens, Erlangen, Germany). The Chronic Kidney Disease Epidemiology Collaboration (CKD-EPI) creatinine equation recommended by the National Kidney Foundation, USA was used to estimate the Glomerular Filtration Rate (eGFR) for the subjects^49,50^.

### High-Resolution Accurate-Mass (HRAM) data analysis

Plasma samples were spiked with an internal standard (IS) of a ^2^H-labelled amino acid mixture (1 ng/mL; U-^2^H labelled amino acid mix >97% purity; Cambridge Isotope Laboratories, Massachusetts, USA), diluted 3-fold with chilled organic solvent (8:1:1, acetonitrile: methanol: acetone) and vortex-mixed. They were then incubated at 4 °C for 30 min and centrifuged at 20000 rcf for 20 min in a refrigerated centrifuge (5810 R, Eppendorf, Eppendorf AG, Hamburg, Germany). Supernatants were transferred to another vial and dried at 40 °C in a vacuum concentrator (Labconco, USA). Metabolites were reconstituted in acetonitrile/water (1:1) and high-throughput, untargeted metabolomics analysis was performed on a high-resolution accurate-mass (HRAM) platform consisting of an ultra-high pressure liquid chromatograph (UHPLC, Thermo Scientific, Vanquish Flex Binary, Waltham, MA, USA) coupled to an orbitrap based mass spectrometer (Q Exactive, Thermo Scientific, San Jose, USA). The mass spectrometer was calibrated by using a positive ion calibration solution (Thermo Scientific^TM^ Pierce LTQ Velos ESI) as an external calibration on daily basis. Separation of the metabolites was achieved by using a Zorbax Eclipse plus-C18 column (150*2.1*1.8 micron, Agilent Technologies, Santa Clara, CA, USA) at 40 °C. The mobile phase was delivered in a reversed-phase gradient elution at 0.35 mL/min, using water (eluent A) and acetonitrile (eluent B), both containing 0.1% formic acid. The following gradient profile was used: 0–3 min: 1% B and increased to 95% B at 14 min and held for 3 min then decreased to 1% B at 17.5 and equilibrated for another 3 min. Reconstituted extracts were loaded on an autosampler where the injection volume was set at 5 μL for each of the solvent blanks, pooled quality control (QC) samples, which included four technical replicates of a pool of aliquots from plasma samples (from each of the study groups,^51^ and study samples in a daily analytical batch.

The mass spectrometer was operated under heated electrospray ionization (HESI-II) positive mode in full scan (m/z 100–1500) and used resolution 70,000 (FWHM) at m/z 200, with automatic gain control (AGC) target of 1 × 10^6^ ions and a maximum ion injection time (IT) of 100 ms. Data-dependent MS/MS were acquired on a “Top5” data-dependent mode using the following parameters: resolution 35,000; AGC 1 × 10^5^ ions; maximum IT 50 ms; 1.0 amu isolation window; combined NCE 25%, 35% and 50% and dynamic exclusion time was set at 10 s. Source ionization parameters were: spray voltage, 3.80 kV; capillary temperature, 330 °C; heater temperature 350 °C and S-Lens level, 50.

Metabolites were visualized, mapped to pathways and automatically identified by *mz*Cloud using Thermo Scientific™ Compound Discoverer™ 3.0 software. An untargeted metabolomics workflow was used to identify the differences in metabolites between samples from the three study groups. This workflow performed the retention time alignment, unknown metabolite detection, metabolite grouping across all samples, predicted elemental compositions for all metabolites, filled gaps across all samples, corrected the chemical background (using blank samples) and normalized the data by using constant mean parameters. QC samples were used for batch normalization and statistical data analysis. Identification of the metabolites was done by using mzCloud (ddMS2) and ChemSpider (formula or exact mass) along with similarity searches for all compounds with ddMS2 data using mzCloud. The identification also applied mzLogic algorithm to rank order ChemSpider results and mapped compounds to biological pathways using Metabolika, BioCys and KEGG (available within Compound Discoverer™ 3.0 software).

### Statistical analyses

Anthropometric, biochemical and dietary data were presented as mean ± SD for normally distributed data or median (quartile1, quartile3) for non-normally distributed data. Normal distributions were examined using the Shapiro-Wilk test and Q-Q plots. Comparison between groups of normally distributed data was performed using ANOVA followed by Tukey’s Post-hoc test and that between non-normally distributed data was performed using Kruskal-Wallis one-way ANOVA followed by Dunn post-hoc test with Bonferroni adjustment for multiple testing. Associations between non-normally distributed data was tested using Spearman’s correlation. *P*-value < 0.05 was considered statistically significant.

For untargeted metabolomic data, groups area ratios, fold change (log_2_ scale), study group-wise coefficient of variance, trend charts, Principal Component Analysis (PCA), as well as differential analysis by ANOVA (per group ratio by ANOVA and TukeyHSD post-hoc test for significance testing adjusted with Benjamini-Hochberg corrections for the false discovery rate) were analysed using the Compound Discoverer™ 3.0 software. Data analyses were conducted using principal component analysis (PCA), an unsupervised method where each point represents an MS spectrum allowing identification of the similarity or the differences between the sample profiles. A framework for assessing specific differences by ANOVA between groups was conducted as follows: first, metabolites were unequivocally identified, confirmed and categorized as significantly different in abundance (dysregulated) between each of the groups (NGT, T2D, and T2D-DN). Next, significant differences in metabolites were sought between the controls and the both diabetes groups, irrespective of their renal function (between NGT and T2D or T2D-DN but similar between T2D and T2D-DN). Next, specific differences were sought for the T2D-DN group (between NGT and T2D-DN and between T2D and T2D-DN but similar between NGT and T2D). Finally, specific differences were sought for the T2D group (between T2D and NGT and between T2D and T2D-DN but similar between NGT and T2D-DN). The FDR adjusted *p-*value was <0.05 for all such metabolites. We used an improved and integrated way of visualizing all the detected metabolites by ‘MetaMapp’ to map all the detected metabolites (with *P* ≤0.05 and fold change ≥1) in network graphs by using KEGG reactant pair database, and Tanimoto similarity-chemical relationship scores^52^. The output from MetaMapp network was further visualized by an open-source platform Cytoscape (version 3.0.) for the metabolite categories that were found to be dysregulated in this study^53^. Each node of the network represents a metabolite or class of metabolites and an edge or line between the nodes denotes a similarity relationship between those metabolites or class of metabolites. The radial layouts were used so that nodes are clustered more tightly if they are more highly interconnected within the network of metabolites for each group.

## Supplementary information

Supplementary material

## Data Availability

The datasets generated and analysed during the current study that support the findings of this study are available from the corresponding author on reasonable request.
