# Supplementary material for: Acyl ethanolamides in Diabetes and Diabetic Nephropathy: Novel targets from untargeted plasma metabolomic profiles of South Asian Indian men
Source: Sci Rep. 2019 Dec 2;9:18117. doi: 10.1038/s41598-019-54584-2 (PMC6889195; doi:10.1038/s41598-019-54584-2)

Supplementary Information

Manuscript title: Acyl ethanolamides in Diabetes and Diabetic Nephropathy: Novel targets from untargeted plasma metabolomic profiles of South Asian Indian men

Sarita Devi[1,#], Bajanai Nongkhlaw[1,#], Limesh M[2], Roshni M. Pasanna[1], Tinku Thomas[3], Rebecca Kuriyan[1], Anura V. Kurpad[1,+] and Arpita Mukhopadhyay[1,+,*]

[1]Division of Nutrition, St John's Research Institute, St John's National Academy of Health Sciences, Bangalore, India;

[2]Department of Nephrology, St John's Medical College and Hospital, St. John's National Academy of Health Sciences, Bangalore, India;

[3]Department of Biostatistics, St John's Medical College and Hospital, St. John's Research Institute, St. John's National Academy of Health Sciences, Bangalore, India.

[#]Joint first authors

[+]Joint senior authors

**Corresponding author:*

Division of Nutrition

Division of Nutrition, St. John's Research Institute, St. John's National Academy of Health Sciences, Sarjapur Road, Bangalore, India. PIN: 560034

Phone: +91-80-49467000

Fax: +91-80-25501088

Email address: arpitam@sjri.res.in

Supplementary Table 1: The 16 metabolites significantly dysregulated between NGT and T2D, T2D and T2D-DN as well as NGT and T2D-DN (FDR adjusted *p*-value <0.05)]. (NGT: normal glucose tolerance, T2D: type 2 diabetes mellitus, T2D-DN: type 2 diabetes mellitus-diabetic nephropathy)

**Metabolite**	**Ratio T2D/NGT**	**Ratio T2D-DN/NGT**	**Ratio T2D-DN/T2D**	**Adjusted *p*-value T2D/NGT**	**Adjusted *p*-value T2D-DN/NGT**	**Adjusted *p*-value T2D-DN/T2D**
(betaS)-beta-Hydroxy-L-tryptophan	3.4	4.9	1.4	0.013	<0.001	0.011
(R)-3-hydroxybutyrylcarnitine	1.5	2.0	1.3	0.001	<0.001	0.008
2-Amino-9,10-epoxy-8-oxodecanoic acid	1.4	1.9	1.3	0.015	<0.001	0.046
6-Oxo-2-piperidinecarboxylic acid	1.5	2.3	1.5	0.001	<0.001	<0.001
Anagestone Acetate	1.4	2.1	1.5	0.029	<0.001	0.005
Apronalide	1.4	2.8	2.0	0.032	<0.001	<0.001
Cholecalciferol	0.7	0.5	0.8	0.006	<0.001	0.043
Diosgenin	0.8	0.6	0.7	0.028	<0.001	0.022
DL-Dipalmitoylphosphatidylcholine	1.5	2.6	1.7	0.029	<0.001	0.002
Imidazolelactate	1.6	3.2	2.0	0.001	<0.001	<0.001
Lauramide	0.8	0.7	0.8	0.023	<0.001	0.031
N-Acetylornithine	1.4	2.5	1.8	0.021	<0.001	<0.001
Primobolan	0.7	0.6	0.8	0.048	<0.001	0.010
Tetrahydrocorticosterone	1.4	3.3	2.3	0.041	<0.001	<0.001
Trigonelline	1.4	2.4	1.7	0.035	<0.001	0.002
Xanthoaphin	0.5	0.1	0.3	0.017	<0.001	<0.001

Supplementary Table 2. The 48 metabolites significantly associated with T2D irrespective of renal function of the subjects [significantly dysregulated (FDR adjusted *p*-value < 0.05) between NGT and T2D and between NGT and T2D-DN but not between T2D and T2D-DN]. (NGT: normal glucose tolerance, T2D: type 2 diabetes mellitus, T2D-DN: type 2 diabetes mellitus-diabetic nephropathy)

**Metabolite**	**Ratio T2D/NGT**	**Ratio T2D-DN/NGT**	**Ratio T2D-DN/T2D**	**Adjusted *p*-value T2D/NGT**	**Adjusted *p*-value T2D-DN/NGT**	**Adjusted *p*-value T2D-DN/T2D**
(2E,4E,14E)-13-Hydroperoxy-N-(2-methylpropyl)icosa-2,4,14-trienamide	0.5	0.5	1.1	0.023	0.013	1.000
18-hydroxy-5Z,8Z,11Z,14Z-eicosatetraenoic acid	0.8	0.7	0.9	0.018	<0.001	1.000
1-Linoleoyl glycerol	1.6	1.6	1.0	0.001	<0.001	1.000
1-Stearoylglycerol	1.7	1.9	1.1	<0.001	<0.001	0.923
2-Linoleoyl glycerol	1.7	1.7	1.0	0.007	<0.001	1.000
Cytidine 5'-diphosphocholine	1.0	1.0	1.0	0.045	0.009	1.000
D-Raffinose	1.6	1.6	1.0	<0.001	<0.001	1.000
Fingolimod	0.5	0.2	0.4	0.035	<0.001	0.285
Jasmonic acid	0.9	1.0	1.0	0.002	0.001	1.000
Palmitoyl sphingomyelin	1.4	1.7	1.2	0.035	<0.001	0.754
Sorbitane trioleate	0.5	0.5	0.9	<0.001	<0.001	0.420
Taurocholic acid	0.3	0.2	0.7	0.042	0.001	1.000
Cassine	0.6	0.6	0.9	<0.001	<0.001	0.938
17α-Methyl-androstan-3-hydroxyimine-17β-ol	0.3	0.3	0.8	<0.001	<0.001	0.188
1-hexadecanoyl-2-(9Z,12Z-octadecadienoyl)-sn-glycero-3-phosphocholine	2.4	2.3	1.0	<0.001	<0.001	1.000
2,3-bis-O-(geranylgeranyl)-sn-glycero-3-phospho-L-serine	1.4	1.3	0.9	0.004	0.001	1.000
2-[(5Z)-5-Tetradecen-1-yl]cyclobutanone	0.4	0.2	0.5	0.031	<0.001	0.134
3-dehydrosphingosine	0.4	0.3	0.9	<0.001	<0.001	0.577
3-Oxalomalic acid	0.9	0.8	0.9	0.008	<0.001	1.000
Diphenyl sulfoxide	1.6	1.4	0.9	<0.001	0.002	0.310
Linoleamide	0.7	0.6	0.9	<0.001	<0.001	0.467
9,10,18-Trihydroxystearic Acid	0.7	0.7	0.9	0.001	<0.001	0.800
Lumichrome	2.3	1.7	0.7	0.028	0.026	1.000
Acetylcholine	0.7	0.8	1.1	<0.001	<0.001	0.599
Acetyl-L-leucyl-L-leucylargininal	1.0	0.9	1.0	0.011	0.001	1.000
Azelnidipine	0.6	0.5	0.9	0.013	0.001	1.000
Bardoxolone methyl	1.4	1.4	1.0	0.033	0.001	1.000
2-Octenoic acid	0.7	0.6	0.8	0.027	<0.001	1.000

4-Oxoretinoic acid	0.6	0.4	0.7	0.001	<0.001	0.220
Cortisol	1.4	1.3	0.9	0.017	0.022	1.000
Drofenine	0.4	0.2	0.6	<0.001	<0.001	0.192
Epicillin	1.2	1.2	1.0	0.017	<0.001	1.000
Epitestosterone	0.7	0.8	1.1	0.011	0.018	0.969
Gamma-Glutamyl-S-allylcysteine	0.7	0.5	0.7	0.049	<0.001	0.160
Glimepiride	1.2	1.2	1.0	0.006	<0.001	1.000
Glycerophospho-N-palmitoyl ethanolamine	1.2	1.3	1.0	0.045	0.002	1.000
Hexadecanamide	0.6	0.4	0.7	0.001	<0.001	0.143
MDMA Methylene homolog	2.1	2.6	1.2	0.038	0.001	1.000
Metformin	835.4	1.5	0.0	<0.001	<0.001	0.098
N-benzoyl-D-arginine-4-nitroanilide	0.5	0.7	1.2	0.001	<0.001	1.000
Oleamide	0.5	0.4	0.8	0.022	<0.001	0.848
Palmitoleoyl ethanolamide	0.4	0.4	0.8	<0.001	<0.001	0.523
PC(18:3(9Z,12Z,15Z)/18:2(9Z,12Z))	2.2	1.7	0.8	<0.001	<0.001	0.686
Stearamide	0.6	0.4	0.7	0.006	<0.001	0.068
Tretinoin	0.4	0.5	1.1	0.003	<0.001	1.000
Yersiniabactin	4.3	3.0	0.7	<0.001	<0.001	0.761
Sphinganine	0.8	0.7	0.9	0.041	<0.001	0.699
γ-Linolenic acid ethyl ester	1.2	1.3	1.0	0.013	0.001	1.000

Supplementary Table 3. The 192 metabolites specifically associated with T2D-DN [significantly dysregulated between NGT and T2D-DN as well as between T2D and T2D-DN (FDR adjusted *p*-value < 0.05) but not between NGT and T2D; and exhibiting a difference of ≥2 folds or ≤0.5 fold between the T2D-DN and T2D groups]. (NGT: normal glucose tolerance, T2D: type 2 diabetes mellitus, T2D-DN: type 2 diabetes mellitus-diabetic nephropathy)

**Metabolite**	**Ratio T2D/NGT**	**Ratio T2D-DN/NGT**	**Ratio T2D-DN/T2D**	**Adjusted *p*-value T2D/NGT**	**Adjusted *p*-value T2D-DN/NGT**	**Adjusted *p*-value T2D-DN/T2D**
12,13-dihydroxy-9Z-octadecenoic acid	1.2	4.9	4.2	1.000	<0.001	<0.001
Pantetheine	0.7	3.7	5.4	1.000	<0.001	<0.001
(2R,3S)-3-Hydroxy-8-methyl-8-azabicyclo[3.2.1]octane-2-carboxylic acid	1.1	2.4	2.1	1.000	<0.001	0.001
(8aR,12S,12aR)-12-Hydroxy-4-methyl-4,5,6,7,8,8a,12,12a-octahydro-2H-3-benzoxecine-2,9(1H)-dione	1.1	7.8	6.8	0.857	<0.001	<0.001
1-(2,4,5-Trimethoxyphenyl)-1,2-propanedione	1.0	2.4	2.3	1.000	<0.001	0.022
1-(2,4-Dihydroxyphenyl)-1-butanone	1.0	3.1	3.0	1.000	<0.001	<0.001
1-(beta-D-ribofuranosyl)thymine	1.1	3.1	2.9	1.000	<0.001	<0.001
1,4,5,6-Tetrahydro-2-methyl-4-pyrimidinecarboxylic acid	1.2	2.7	2.2	0.207	<0.001	<0.001
N-oleoyl tyrosine	0.7	0.4	0.5	0.599	<0.001	0.001
15-(tert-Butyl)-2,3,5,6,8,9,11,12-octahydro-1,4,7,10,13-benzopentaoxacyclopentadecine	0.6	1.6	2.6	0.440	0.030	<0.001
16α-Hydroxydehydroepiandrosterone	0.7	3.0	4.2	0.650	<0.001	<0.001
19(R)-HETE	1.2	2.9	2.4	1.000	<0.001	<0.001
19-Nortestosterone	1.0	2.5	2.4	1.000	<0.001	0.009
1-alpha,24R,25-Trihydroxyvitamin D2	0.8	0.4	0.4	0.385	<0.001	0.003
1-Naphthol	1.1	2.3	2.1	0.493	<0.001	0.009
1-Pentofuranosyl-2,4(1H,3H)-pyrimidinedione	1.0	2.5	2.5	1.000	<0.001	<0.001
2-({2-[4-(4-Fluorophenyl)piperazino]-2-oxoethyl}sulfanyl)acetic acid	1.5	0.5	0.4	1.000	0.006	<0.001
2-(1H-Benzimidazol-2-ylsulfanyl)-N-(3-cyano-6-methyl-4,5,6,7-tetrahydro-1-benzothiophen-2-yl)acetamide	1.0	5.8	5.8	0.458	<0.001	<0.001
2-(Phenylsulfonyl)acetophenone	1.1	2.2	2.0	1.000	0.001	0.001
2,6-quinolinediol	1.3	3.9	3.0	0.410	<0.001	<0.001
2-[2-(3,4-dimethoxyphenyl)ethyl]-4-methoxy-2,3-dihydropyran-6-one	0.9	2.0	2.1	1.000	<0.001	<0.001
2-Aminoadenosine	0.9	3.2	3.4	1.000	<0.001	<0.001
2-Hydroxyhippuric acid	1.4	3.4	2.4	0.422	<0.001	0.040
2-methoxyacetaminophen sulfate	0.9	2.1	2.4	0.979	<0.001	<0.001

2-methylbutyrylcarnitine	1.1	0.5	0.5	1.000	<0.001	<0.001
3-(2-Hydroxy-5-methoxybenzoyl)-2-(4-methylphenyl)isoindolin-1-one	0.8	0.4	0.4	1.000	<0.001	<0.001
3,13,13,17-Tetramethyl-21-oxa-12-azahexacyclo[10.7.1.1~2,17~.0~5,20~.0~6,11~.0~14,19~]henicosa-1(20),2,4,6,8,10-hexaen-16-ol	0.9	0.4	0.5	0.798	<0.001	0.001
3,4,5-trimethoxydihydrocinnamic acid	1.0	2.9	2.8	1.000	0.027	0.037
3',4'-Dimethoxyacetophenone	1.0	2.8	2.9	1.000	0.035	0.039
3-Hydroxy-cis-5-tetradecenoylcarnitine	0.9	2.1	2.2	1.000	<0.001	<0.001
4-methoxy-5-methyl-6-[(2S,3S)-2-methyl-3-[(2Z,4E)-4-methyl-5-[(1S,2S,4R,5R)-1,2,4-trimethyl-3,6-dioxabicyclo[3.1.0]hexan-4-yl]penta-2,4-dien-2-yl]oxiran-2-yl]pyran-2-one	1.0	3.3	3.2	1.000	<0.001	<0.001
Alpha-Farnesene	1.2	0.5	0.4	1.000	<0.001	<0.001
5-(6-Hydroxy-2,5,7,8-tetramethyl-chroman-2-yl)-2-methyl-pentanoic acid	1.0	2.9	3.0	1.000	<0.001	<0.001
5-Butyl-1,3-oxazole	1.5	3.3	2.2	0.101	<0.001	<0.001
5-guanidino-2-oxopentanoic acid	1.1	2.4	2.1	1.000	<0.001	<0.001
5-Indolol	1.2	3.7	3.2	0.472	<0.001	<0.001
5α-Dihydrotestosterone	0.9	1.8	2.0	1.000	0.003	<0.001
5α-Tetrahydrocortisol	0.7	9.2	12.3	1.000	<0.001	<0.001
2,2'-[4-(2-Hydroxyethylamino)-3-nitrophenylimino]diethanol	1.0	4.6	4.4	1.000	<0.001	<0.001
6-Hydroxy-5-methyl-4,11-dioxoundecanoic acid	1.1	2.9	2.6	1.000	<0.001	<0.001
6-Ketoprostaglandin F1α	0.7	1.7	2.3	0.923	<0.001	<0.001
6β-Hydroxyfluoxymesterone	1.4	3.4	2.4	0.435	<0.001	<0.001
[2-(Dimethoxymethyl)-1-heptenyl]benzene	0.9	2.4	2.8	1.000	<0.001	<0.001
N-Nitrosodibutylamine	0.9	2.2	2.4	1.000	<0.001	<0.001
Noroxycodone	0.5	0.1	0.2	1.000	<0.001	0.001
9-O-Demethyl-2alpha-hydroxyhomolycorine	0.7	0.3	0.5	1.000	0.028	0.022
Acamprosate	1.1	2.5	2.3	1.000	<0.001	<0.001
Acetyl-L-carnitine	1.4	4.3	3.1	0.280	<0.001	<0.001
Acetyllycopsamine	1.2	3.0	2.5	1.000	<0.001	<0.001
Actinonin	0.8	1.7	2.1	1.000	0.001	0.010
Aderbasib	1.2	3.3	2.7	1.000	<0.001	<0.001
Allixin	1.1	2.6	2.5	1.000	<0.001	<0.001
Alloxydim	1.0	2.7	2.7	0.799	<0.001	<0.001
Alminoprofen	1.1	0.4	0.4	1.000	<0.001	<0.001
Aloesin	1.0	2.1	2.2	1.000	0.002	0.001
Alpha-(4-methoxyphenyl)-6-methyl-2-pyridineacrylic acid	1.0	2.2	2.1	1.000	<0.001	<0.001

Alpha-Cehc	1.0	2.0	2.0	1.000	<0.001	<0.001
Amidapsone	1.0	4.4	4.6	1.000	<0.001	<0.001
Amygdalin	0.8	1.6	2.0	1.000	0.024	0.019
Andrographolide	0.7	2.1	3.0	1.000	<0.001	<0.001
Androsterone	1.5	3.5	2.4	0.053	<0.001	<0.001
Apiol	1.0	2.9	2.8	1.000	0.027	0.037
Arachidonic acid	1.3	2.7	2.0	0.538	<0.001	<0.001
Artemisinin	1.1	2.4	2.2	0.659	<0.001	<0.001
Atractylone	1.2	3.5	2.9	0.642	<0.001	<0.001
Benfluralin	0.9	11.3	12.0	0.276	<0.001	<0.001
Benznidazole	0.6	2.5	3.9	1.000	<0.001	<0.001
Betaine	1.1	2.3	2.1	1.000	<0.001	<0.001
Buflomedil	1.3	3.3	2.6	0.654	<0.001	0.005
Butabarbital	1.1	2.6	2.4	1.000	<0.001	0.001
Callystatin A	0.9	0.4	0.4	1.000	<0.001	0.021
Candoxatril	1.2	2.6	2.3	1.000	0.004	0.028
Cannabidivarin	1.0	2.1	2.1	1.000	<0.001	<0.001
Carbaprostacyclin	1.4	3.4	2.4	0.075	<0.001	<0.001
Carisoprodol	1.3	3.4	2.6	0.658	<0.001	<0.001
Carteolol	1.0	3.1	3.2	1.000	<0.001	<0.001
Cinnamoylglycine	0.8	4.1	5.2	1.000	<0.001	<0.001
Ciprostene	0.9	0.4	0.4	1.000	<0.001	<0.001
Clethodim sulfone	1.2	2.8	2.4	0.946	<0.001	<0.001
Clonazolam	1.0	2.5	2.6	1.000	<0.001	<0.001
Codeine	1.0	0.3	0.3	0.932	<0.001	0.004
Coenzyme Q2	0.9	2.1	2.2	1.000	<0.001	<0.001
Coronatine	1.0	3.1	3.0	1.000	<0.001	<0.001
Cyclooctatin	1.9	4.8	2.5	0.182	<0.001	<0.001
Glyol	1.2	3.5	3.0	1.000	<0.001	<0.001
Desonide	0.5	3.4	7.2	1.000	0.001	<0.001
Dihydroberberine	1.3	0.6	0.4	1.000	0.002	0.017
Dihydrocodeine	1.0	4.4	4.3	1.000	<0.001	<0.001
DL-Glutamine	1.3	7.7	5.9	0.500	<0.001	<0.001
D-Maltose	1.0	3.6	3.6	0.970	<0.001	<0.001
Docosahexaenoic acid ethyl ester	0.8	3.5	4.1	1.000	<0.001	<0.001
Docosapentaenoic acid	1.2	0.6	0.5	1.000	0.008	0.015
Doramectin	1.1	5.0	4.8	1.000	<0.001	<0.001

D-α-Tocopherol	1.0	0.5	0.5	1.000	<0.001	0.003
7-Methylxanthine	0.9	1.9	2.1	1.000	<0.001	<0.001
Epitestosterone glucuronide	0.7	3.6	5.1	1.000	<0.001	<0.001
Europine	0.8	5.3	6.7	1.000	<0.001	<0.001
Theasapogenol B	0.9	0.2	0.2	0.262	<0.001	<0.001
Salinosporamide B	1.5	3.6	2.5	1.000	<0.001	<0.001
Glycerol tripropionate	1.1	4.0	3.6	1.000	<0.001	<0.001
Flossonol	1.2	2.6	2.3	0.510	<0.001	<0.001
Gamma-glutamyltyramine	1.1	3.0	2.9	1.000	<0.001	<0.001
Genistin	0.9	0.3	0.3	1.000	<0.001	0.006
Glaucarubinone	0.9	2.0	2.3	1.000	<0.001	<0.001
Hexanoylcarnitine	1.3	2.8	2.2	1.000	<0.001	<0.001
Hippuric acid	1.0	2.1	2.2	1.000	<0.001	0.001
Homocitrulline	0.9	2.3	2.5	0.807	<0.001	<0.001
Hostmaniane	1.2	2.4	2.1	0.739	<0.001	<0.001
Hydroxypropionylcarnitine	0.9	3.1	3.6	1.000	<0.001	<0.001
Indane	1.2	0.5	0.4	1.000	0.011	0.019
Indol-2-one	0.8	3.8	4.9	1.000	<0.001	<0.001
Indol-4-one	1.0	3.2	3.1	1.000	<0.001	<0.001
Indolyl-3-acryloylglycine	1.2	2.8	2.3	1.000	0.026	0.006
Iobitridol	0.8	6.2	7.5	1.000	<0.001	<0.001
Kanamycin	1.1	6.3	6.0	0.840	<0.001	<0.001
L-Ergothioneine	1.4	0.7	0.5	0.726	<0.001	<0.001
Leucine	1.1	2.8	2.7	1.000	<0.001	<0.001
Leucylproline	1.0	2.1	2.1	1.000	<0.001	<0.001
L-Glutathione oxidized	1.0	2.5	2.3	1.000	<0.001	<0.001
Lincomycin	1.2	3.1	2.6	0.784	<0.001	<0.001
L-Kynurenine	1.0	2.1	2.0	1.000	<0.001	<0.001
L-Pyroglutamic acid	1.5	3.7	2.4	0.292	<0.001	<0.001
L-Tyrosine	1.0	2.0	2.1	1.000	<0.001	<0.001
Magnolol	0.7	1.8	2.5	1.000	0.001	<0.001
Mephenytoin	1.5	5.4	3.7	0.351	<0.001	<0.001
Metharbital	1.3	3.5	2.8	0.440	<0.001	<0.001
Methasterone	0.9	1.7	2.0	1.000	<0.001	<0.001
Methionine	1.8	12.5	6.9	0.213	<0.001	<0.001
Methyl (3-hydroxy-2-oxo-2,3-dihydro-1H-indol-3-yl)acetate	0.6	3.1	5.0	1.000	0.012	0.002

Methyl indole-3-acetate	1.1	3.5	3.3	1.000	<0.001	<0.001
Milbemycin A4 oxime	0.9	1.9	2.0	1.000	<0.001	<0.001
Miltirone	0.5	3.4	6.9	1.000	<0.001	<0.001
Morphinone	0.7	0.2	0.2	1.000	<0.001	0.001
Myriocin	0.8	1.6	2.1	1.000	<0.001	<0.001
N-(4-coumaroyl)-L-homoserine lactone	1.5	8.3	5.5	0.526	<0.001	<0.001
N-(indol-3-ylacetyl)glutamine	1.5	5.4	3.5	0.944	<0.001	<0.001
N-(indole-3-acetyl)glutamic acid	1.0	2.3	2.2	1.000	<0.001	<0.001
N-[(9-beta-D-ribofuranosylpurin-6-yl)carbamoyl]threonine	0.9	2.6	2.8	1.000	<0.001	<0.001
N1-(2-Pyridylmethyl)-4-methylbenzene-1-sulfonamide	1.4	3.2	2.3	0.453	<0.001	<0.001
N4-Acetyl sulfamethazine	0.7	5.5	7.8	1.000	<0.001	<0.001
N-Acetylhistamine	0.8	2.6	3.3	1.000	0.011	0.005
N-Acetylvaline	1.1	2.4	2.1	1.000	<0.001	<0.001
Nalorphine	0.7	0.2	0.2	0.519	<0.001	0.002
N-Butyl salicylate	1.2	2.3	2.0	0.619	<0.001	0.002
N-Caffeoylputrescine	1.1	3.9	3.6	1.000	<0.001	<0.001
N-Phenylacetylglutamic acid	0.8	2.0	2.5	1.000	<0.001	<0.001
N-Phenylacetylglutamine	1.4	8.0	5.8	0.391	<0.001	<0.001
O-3-methylglutarylcarnitine	1.2	3.1	2.6	0.480	<0.001	<0.001
Oleyl anilide	1.0	2.3	2.3	1.000	<0.001	<0.001
Ophthalmic acid	1.1	2.7	2.5	0.863	<0.001	<0.001
Ornithinoalanine	1.4	4.0	2.8	0.496	<0.001	<0.001
Palmitoylcarnitine	0.8	0.1	0.1	1.000	0.001	0.008
Pantoprazole	1.0	2.0	2.0	0.460	<0.001	0.003
Phenisopham	1.3	10.2	7.8	0.889	<0.001	<0.001
Piperanine	1.2	0.4	0.3	1.000	0.007	0.003
Piperine	0.7	0.2	0.2	1.000	<0.001	0.001
Prednisone	0.9	2.2	2.5	1.000	<0.001	<0.001
Pregnanetriol	0.9	2.1	2.3	1.000	<0.001	<0.001
Pregnenolone	0.9	1.9	2.0	1.000	0.001	0.001
Proacaciberin	1.0	2.3	2.3	1.000	<0.001	<0.001
Probenazole	0.9	2.3	2.5	1.000	<0.001	<0.001
Progesterone	1.3	3.0	2.2	0.169	<0.001	<0.001
Promolate	1.0	3.0	2.9	1.000	<0.001	<0.001
Propionylcarnitine	1.0	2.0	2.0	1.000	<0.001	<0.001
Pulcherriminic acid	0.9	2.6	2.7	1.000	<0.001	<0.001

Pumiliotoxin 251D	0.7	1.5	2.3	0.325	0.001	<0.001
Ricinoleic Acid	0.8	0.4	0.5	0.511	<0.001	0.002
Roxatidine	1.1	8.1	7.4	1.000	<0.001	<0.001
Ryania	0.5	3.5	7.3	1.000	<0.001	<0.001
Salinomycin	0.6	1.5	2.4	0.539	0.013	<0.001
Secobarbital	1.0	2.1	2.2	1.000	<0.001	<0.001
Sesamin	1.2	9.4	7.9	0.513	<0.001	<0.001
Siaresinol	1.0	0.2	0.2	0.265	<0.001	<0.001
Soyasaponin I	1.4	3.5	2.4	0.599	<0.001	<0.001
Stylisterol B	0.8	0.2	0.3	0.314	<0.001	0.001
Sucrose acetate isobutyrate	1.0	2.7	2.7	1.000	<0.001	<0.001
Telmisartan	0.8	3.2	3.9	0.188	<0.001	0.027
Tetrahydrocortisone	1.0	3.1	3.2	1.000	<0.001	<0.001
Tetranor 12-HETE	0.7	1.5	2.0	0.811	0.004	<0.001
thr-tyr	1.2	3.6	2.9	0.998	<0.001	<0.001
2-Tetrahydrothiopheneacetic acid	1.2	2.6	2.2	0.985	<0.001	<0.001
N-Acetyl-L-tryptophan	1.0	2.7	2.6	1.000	0.007	0.001
Lophophorine	1.2	2.7	2.2	0.979	<0.001	<0.001
N-Acetylthreonine	1.0	2.6	2.5	1.000	<0.001	<0.001
Valyltyrosine	1.1	2.3	2.2	1.000	<0.001	<0.001
Valylvaline	1.5	3.7	2.5	1.000	<0.001	<0.001
Veliflapon	0.8	0.3	0.3	0.617	<0.001	<0.001
Zalcitabine	0.8	2.2	2.6	1.000	<0.001	<0.001
Zopiclone	1.1	7.9	7.4	0.918	<0.001	<0.001
α-Hydroxyhippuric acid	0.9	6.0	6.7	1.000	<0.001	<0.001
α-Zearalanol	1.0	5.1	5.0	0.094	<0.001	0.012
β-Cortolone	1.0	2.3	2.3	1.000	<0.001	<0.001

Supplementary Table 4. Spearman's correlation between renal function, expressed as estimated Glomerular Filtration Rate (eGFR), and the 20 metabolites with least FDR adjusted *p*-value in difference in abundance between T2D and T2D-DN. (NGT: normal glucose tolerance, T2D: type 2 diabetes mellitus, T2D-DN: type 2 diabetes mellitus-diabetic nephropathy)

**Metabolite**	**eGFR (mL/min/1.73m^2)**					
	**NGT**		**T2D**		**T2D-DN**	
	**rho**	***p*-value**	**rho**	***p*-value**	**rho**	***p*-value**
Imidazolelactate	-0.20	0.286	-0.42	0.025	-0.62	<0.001
L-Pyroglutamic acid	-0.30	0.107	-0.29	0.129	-0.60	<0.001
Mephenytoin	-0.26	0.163	-0.14	0.474	-0.66	<0.001
N-Phenylacetylglutamine	-0.31	0.094	-0.29	0.131	-0.61	<0.001
N-Phenylacetylglutamic acid	-0.33	0.080	-0.39	0.037	-0.64	<0.001
2,6-quinolinediol	0.11	0.575	-0.55	0.002	-0.70	<0.001
DL-Glutamine	-0.30	0.106	-0.26	0.167	-0.60	<0.001
Ornithinoalanine	-0.33	0.079	-0.24	0.207	-0.65	<0.001
Secobarbital	-0.41	0.027	-0.21	0.267	-0.71	<0.001
α-Hydroxyhippuric acid	0.15	0.444	-0.13	0.505	-0.63	<0.001
N-Acetylthreonine	-0.11	0.571	0.11	0.567	-0.53	0.002
N-Caffeoylputrescine	-0.05	0.783	-0.10	0.599	-0.74	<0.001
N-[(9-beta-D-ribofuranosylpurin-6-yl)carbamoyl]threonine	-0.42	0.022	-0.38	0.042	-0.79	<0.001
Indol-4-one	-0.26	0.158	-0.08	0.677	-0.59	<0.001
Glycerol tripropionate	-0.23	0.223	-0.20	0.298	-0.59	<0.001
EUROPINE	-0.12	0.528	-0.18	0.355	-0.72	<0.001
Aderbasib	-0.21	0.259	-0.19	0.321	-0.59	0.001
[2-(Dimethoxymethyl)-1-heptenyl]benzene	-0.03	0.884	-0.50	0.006	-0.60	<0.001
1-(beta-D-ribofuranosyl)thymine	0.24	0.208	-0.43	0.019	-0.69	<0.001
2-methoxyacetaminophen sulfate	-0.40	0.032	0.00	0.994	-0.68	<0.001

Supplementary Table 5. The 3 metabolites specifically associated with T2D [significantly dysregulated between NGT and T2D as well as between T2D and T2D-DN (FDR adjusted *p*-value < 0.05) but not between NGT and T2D-DN. (NGT: normal glucose tolerance, T2D: type 2 diabetes mellitus, T2D-DN: type 2 diabetes mellitus-diabetic nephropathy)

**Metabolite**	**Ratio T2D/NGT**	**Ratio T2D-DN/NGT**	**Ratio T2D-DN/T2D**	**Adjusted *p*-value T2D/NGT**	**Adjusted *p*-value T2D-DN/NGT**	**Adjusted *p*-value T2D-DN/T2D**
1-arachidonoyl-sn-glycero-3-phosphocholine	1.5	1.0	0.6	0.015	0.859	0.035
4-(8-Methyl-8,9-dihydro-7H-[1,3]dioxolo[4,5-h][2,3]benzodiazepin-5-yl)aniline	1.4	0.9	0.7	0.044	1.000	0.004
Phenylalanylproline	0.7	1.1	1.6	0.009	1.000	<0.001

Supplementary Figure 1. Box plots depicting levels of (a) imidazolelactate, (b) cholecalciferol (c) 1-Stearoylglycerol, (d) palmitoylcarnitine, (e) choline, (f) 1-arachidonoyl-sn-glycero-3-phosphocholine, (g) phenylalanylproline and (h) 4-(8-Methyl-8,9-dihydro-7H-[1,3]dioxolo[4,5-h][2,3]benzodiazepin-5-yl)aniline in normal glucose tolerance (dark blue), type 2 diabetes mellitus (orange) and type 2 diabetes mellitus-diabetic nephropathy (light blue) subjects.

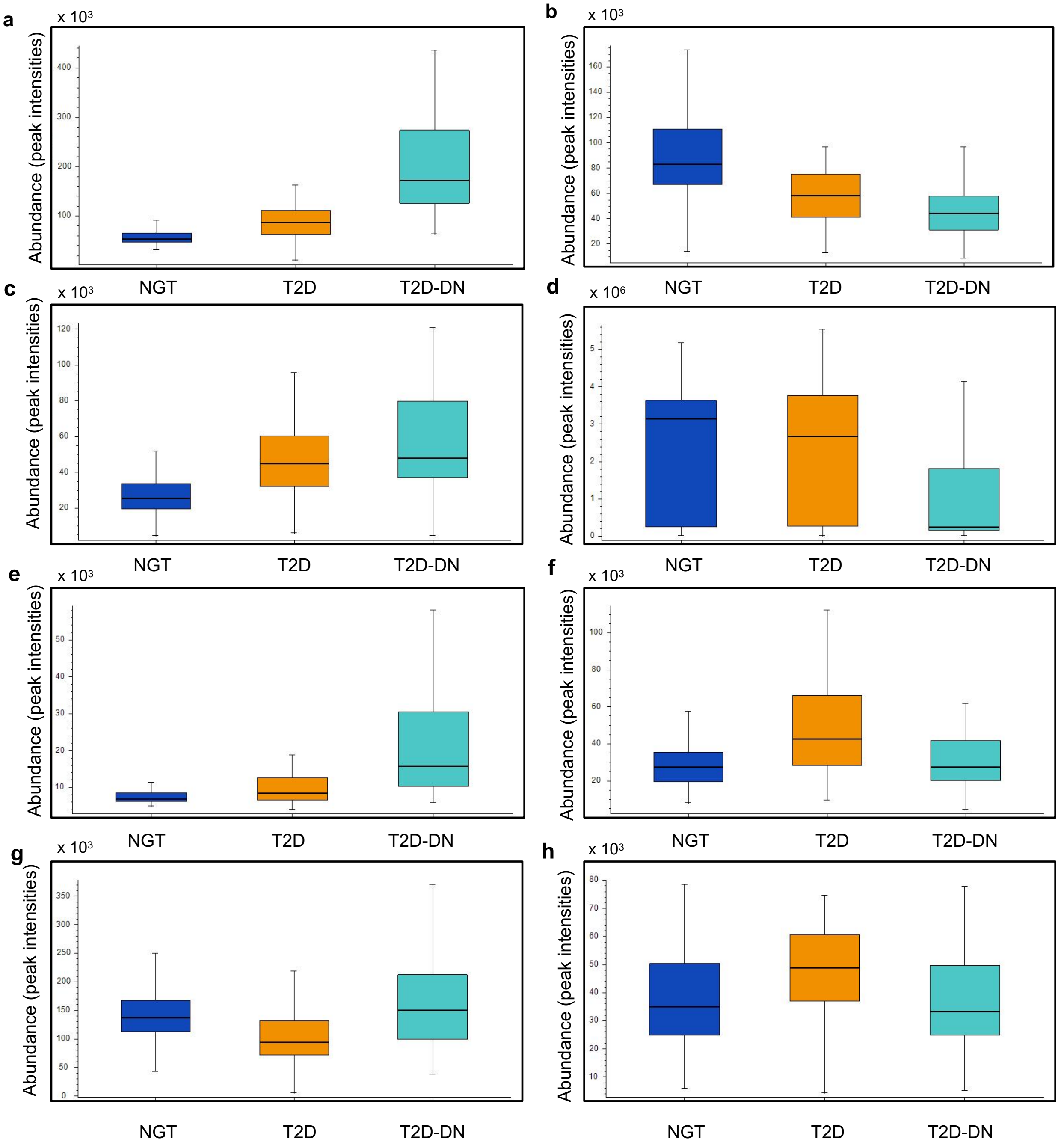

Supplementary Figure 2. Biochemical pathway and chemical relationships network of the 192 metabolites specifically associated with T2D-DN [significantly dysregulated between NGT and T2D-DN as well as between T2D and T2D-DN (FDR adjusted *P* value < 0.05; and exhibiting a difference in ratio of ≥2 or ≤0.5 between the T2D-DN and T2D groups) but not between NGT and T2D]. Blue represents downregulated and red represents upregulated metabolites in T2D-DN compared to T2D and to NGT. (NGT: normal glucose tolerance, T2D: type 2 diabetes mellitus, T2D-DN: type 2 diabetes mellitus-diabetic nephropathy

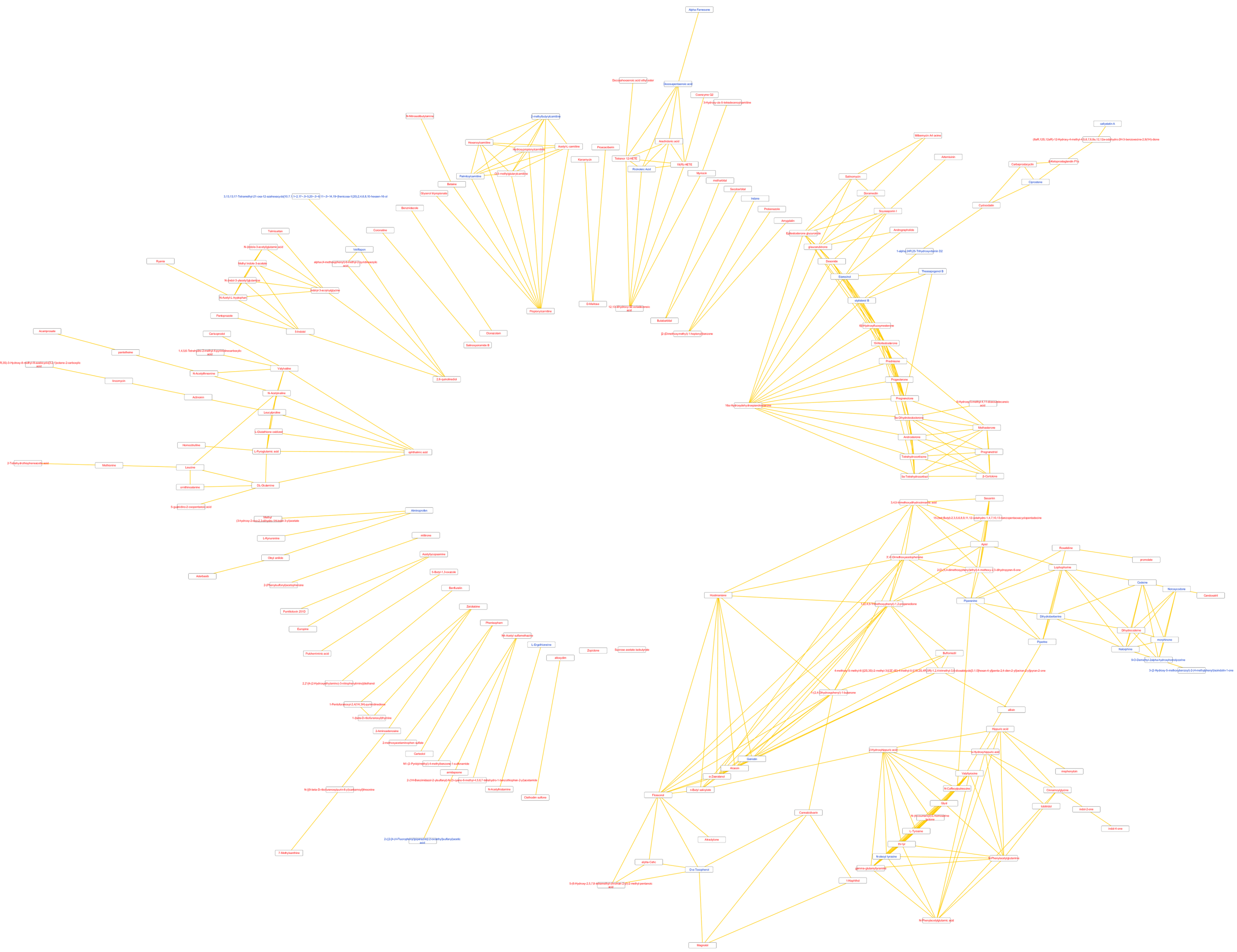

Supplement: Supplementary file 1 — Supplementary material [file 41598_2019_54584_MOESM1_ESM.pdf]
